# Risk Factors for Early Neonatal Hypocalcemia in Preterm Neonates Born After 32 Weeks Gestation

**DOI:** 10.3390/children12091213

**Published:** 2025-09-10

**Authors:** Jelena Sabljić, Edita Runjić, Klara Čogelja, Blagoja Markoski, Marijana Barbača, Boris Bačić

**Affiliations:** 1 Department of Gynaecology and Obstetrics, Division of Neonatology, University Hospital of Split, 21 000 Split, Croatia; jsabljic@kbsplit.hr (J.S.); kcogelja@kbsplit.hr (K.Č.); 2 School of Medicine, University of Split, 21 000 Split, Croatia; mb71921@mefst.hr (M.B.); bbacic@kbsplit.hr (B.B.); 3 Department of Paediatric Diseases, Division of Endocrinology and Neurology, University Hospital of Split, 21 000 Split, Croatia; 4 Department of Gynaecology and Obstetrics, Division of Gynaecologic Oncology and Urology, University Hospital of Split, 21 000 Split, Croatia; bmarkoski@kbsplit.hr

**Keywords:** hypocalcemia, neonate, fetal growth restriction, cesarean section, cardiotocography, acid–base balance, blood gas analysis

## Abstract

**Highlights:**

**What are the main findings?**

- Fetal growth restriction reduces and cesarean delivery increases the probability of early neonatal hypocalcemia in moderate and late neonates.
- Cardiotocography (three-tiered fetal heart rate categorization) and neonatal intensive care (NICU) admission acid base and blood gas analysis are not risk factors for early neonatal hypocalcemia.

**What is the implication of the main finding?**

- Neonates born by cesarean delivery should be screened for early neonatal hypocalcemia.
- Fetal growth restriction could modulate the effect of perinatal hypoxia on neonatal calcium levels.

**Abstract:**

**Background/Objectives**: Early neonatal hypocalcemia is a common metabolic disorder in premature neonates with various risk factors, including perinatal asphyxia and fetal growth restriction (FGR). We aimed to investigate the incidence of early neonatal hypocalcemia in preterm neonates with and without FGR and to explore several maternal and neonatal risk factors for early neonatal hypocalcemia. Cardiotocography (three-tiered fetal heart rate categorization) was a novel risk factor. **Materials and methods:** This was a secondary analysis of the retrospective, single-center, case-control study of neonates admitted to a neonatal intensive care unit (NICU) between January 2021 and December 2023. The study included 24 neonates with FGR and 124 control neonates without FGR born at 33 to 36 6/7 gestational weeks. **Results:** Total serum Ca was significantly lower in control neonates (2.042 (SD 0.208)) compared to neonates with FGR (2.178 (SD 0.180)) (*p* = 0.004), and early neonatal hypocalcemia was significantly higher in control neonates (42.75%) compared to neonates with FGR (4.35%) (*p* < 0.001). There was no statistical difference in acid base and blood gas analysis between FGR and control (*p* > 0.05). Logistic regression with the backward method showed that FGR reduces the probability of early neonatal hypocalcemia by 96.3% (t = 9.679, *p* = 0.001), and cesarean delivery increases it by 2.702 times (t = 6.963, *p* = 0.004). **Conclusions:** In this observational study, FGR was found to reduce and cesarean delivery was found to increase the probability of early neonatal hypocalcemia in moderate and late neonates. Clinicians should consider screening neonates born by cesarean delivery for early neonatal hypocalcemia. Three-tiered fetal heart rate categorization and acid base and blood gas analysis upon NICU admission cannot alert neonatologists to early neonatal hypocalcemia.

## 1. Introduction

Early neonatal hypocalcemia is a common metabolic disorder in preterm neonates, typically presenting within the first three days of life. Decades ago, perinatal asphyxia was recognized as a risk factor for the development of early neonatal hypocalcemia [1,2,3]. Further studies confirmed lower serum calcium levels in asphyxiated neonates [4,5,6,7]. The main causes of hypocalcemia in neonates with asphyxia include increased phosphate load due to cellular damage, increased calcitonin production, renal failure, and decreased parathyroid hormone (PTH) secretion [1,8,9]. Early identification of neonatal hypocalcemia is essential for timely calcium replacement and prevention of symptomatic clinical presentations.

Neonates with fetal growth restriction were associated with early neonatal hypocalcemia, mostly reporting increased early neonatal hypocalcemia as the severity of fetal growth restriction progressed [8,10,11,12,13,14]. More recent data about fetal growth restriction in premature neonates as a risk factor for early neonatal hypocalcemia are scarce. Recently, Menegelli et al. reported that neonates with fetal growth restriction had higher serum calcium levels at birth compared to neonates without fetal growth restriction in a study of bone status and early nutrition on 150 preterm neonates <34 gestational weeks [15]. Kolsi et al. reported that prematurity (74%) was the most common cause of early neonatal hypocalcemia, and it was a combination of several causes, including perinatal asphyxia (26%) and fetal growth restriction (19%) [16].

Cardiotocography is a method with the main purpose of detecting fetal hypoxia. Despite various limitations, cardiotocography provides us with data about fetal heart rate, and it is often analyzed as a predictor of various neonatal outcomes [17,18]. Three-tiered fetal heart rate (FHR) categorization is among the most used intrapartum electronic fetal monitoring methods. Category I FHR tracings are normal, category II FHR traces are indeterminate, and category III tracings are abnormal [19,20]. Umbilical cord blood gas and acid–base assessment are the most objective determinants of the fetal metabolic condition at the moment of birth [21].

Due to various factors that can influence early neonatal hypocalcemia, we aimed to analyze maternal and neonatal factors associated with its development in moderate and late preterm neonates. Presuming that cardiotocography and admission acid–base and blood gas could indicate perinatal hypoxia/asphyxia, we included them as predictors of early neonatal hypocalcemia. To our knowledge, this is the first study to include cardiotocography as a predictor of early neonatal hypocalcemia. We aimed to investigate the differences between moderate and late neonates with and without fetal growth restriction in terms of early neonatal hypocalcemia, hypoglycemia during the first day of life, and acid base and blood gas parameters upon neonatal intensive care unit (NICU) admission.

## 2. Materials and Methods

The present study is a secondary data analysis of data from the retrospective single-center case-control study investigating the outcome of preterm neonates > 32 weeks gestation in relation to three-tiered fetal heart rate categorization that included 25 neonates with FGR as cases and 131 neonates without FGR as controls. This study was conducted at the Department of Gynaecology and Obstetrics, Division of Neonatology, University Hospital of Split, Split, Croatia. Maternal and neonatal data were retrieved manually by study personnel from the center’s computerized database and the center’s paper medical records. The retrieved data included maternal and neonatal characteristics, perinatal medical history, and discharge letters, respectively. A detailed methodology of the primary study was published elsewhere [22].

### 2.1. Early Neonatal Hypocalcemia and Neonatal Hypoglycemia

For the present study, data about total serum Ca or ionized Ca levels, serum glucose level, acid base, and blood gas were included in the analysis. Early neonatal hypocalcemia was defined as total serum calcium < 8 mg/dL (2 mmol/L) or ionized calcium < 4.4 mg/dL (1.1 mmol/L) for term or preterm neonates weighing > 1500 g at birth and total serum calcium < 7 mg/dL (1.75 mmol/L) or ionized calcium < 4 mg/dL (1 mmol/L) for very low birth weight neonates weighing < 1500 g [8]. Neonatal hypoglycemia was defined as a glucose level below 2.6 mmol/L during the first day of life [23].

### 2.2. Inclusion Criteria

Inclusion criteria for this present study included at least one measurement of calcium level (total serum or ionized) during the first three days of life, serum glucose level, and admission acid–base and blood gas analysis during the first day of life. In the cases where multiple glucose measurements were taken during the first day of life, the lowest serum glucose level was analyzed. In our NICU, the standard procedure after neonatal admission is routine analysis of the venous acid–base balance and blood gas using an automatic blood gas analyzer. Blood samples were collected from umbilical vein or peripheral veins. Routine acid base and blood gas data included pH, pCO_2_, HCO_3_ standard (st), base excess (BE) in extracellular fluid, and sO_2_. One neonate with FGR and seven neonates without FGR were excluded due to missing data for this study. A total of 24 FGR and 124 control neonates born at 33 to 36 6/7 gestational weeks were included in the present study.

### 2.3. Statistical Analysis

The statistical analysis of data was conducted by software SPSS 26, producer IBM, Chicago, IL, USA. Variables were tested for normal distribution by applying the Kolmogorov–Smirnof test before applying all subsequent statistical tests. Categorical variables were reported as frequencies and percentages, while descriptive data were presented as mean and standard deviation (SD) or as the median and interquartile range (IQR) for non-normally distributed data. Student *t*-tests, Chi-square tests, and Mann–Whitney tests were conducted for total serum Ca level, serum glucose level, early neonatal hypocalcemia, neonatal hypoglycemia, acid–base, and blood gas differences between neonates with and without FGR. A logistic regression model with the backward method was used to identify maternal and neonatal variables associated with early neonatal hypocalcemia. Statistical significance was set at *p* < 0.05 for all tests.

## 3. Results

### 3.1. Difference in Early Neonatal Hypocalcemia, Hypoglycemia, Acid Base and Blood Gas Analysis Between Neonates with and Without FGR

One neonate with FGR (1.16) and four neonates without FGR (M 1.11, SD 0.07) had only their ionized Ca levels measured, without total serum Ca, in the first three days of life. Due to the small number of neonates with ionized Ca levels, we were not able to statistically test the difference, but they were included in early neonatal hypocalcemia according to the inclusion diagnostic criteria for early neonatal hypocalcemia. Total serum Ca levels were statistically significantly higher in neonates with FGR compared to neonates without FGR (*p* = 0.004) (Table 1). Early neonatal hypocalcemia was statistically significantly less likely to occur in the neonates with FGR compared to neonates without FGR (4.35% vs. 42.75%, *p* > 0.001) (Table 1). There was no statistically significant difference in serum glucose levels and hypoglycemia between the groups (*p* = 0.334, *p* = 0.455) (Table 1). There was no statistically significant difference in pH, pCO_2_, HCO_3_ (st), BE (ef), and sO_2_ between neonates with and without FGR (*p* > 0.05) (Table 2). The overall rate of neonatal hypoglycemia in the first 24 h was 31.3%, and the overall rate of early neonatal hypocalcemia was 36.5%.

### 3.2. Logistic Regression Analysis of Predictors of Early Neonatal Hypocalcemia

Logistic regression with the backward method showed that fetal growth restriction reduced the probability of having early neonatal hypocalcemia by 96.3% (t = 9.679, *p* = 0.001) (Table 3). Cesarean delivery increased the probability for early neonatal hypocalcemia by 2.702 times (t = 6.963, *p* = 0.004).

## 4. Discussion

Our study reported a higher frequency of early neonatal hypocalcemia in neonates without fetal growth restriction compared to neonates with fetal growth restriction born at 33 to 36 6/7 gestational weeks. That is consistent with Meneghelli’s study data, which showed statistically significant higher serum calcium levels at birth in neonates with fetal growth restriction compared to neonates without fetal growth restriction. They proposed a potential interpretation that, in the case of hypophosphatemia, phosphorus is released from the bone, and therefore, calcium is also mobilized, leading to higher calcium levels [15,24]. In a descriptive retrospective single-center study identifying neonates with early neonatal hypocalcemia, Kolsi et al. reported that prematurity was the main cause (74%) of early neonatal hypocalcemia, followed by perinatal asphyxia (26%), maternal diabetes (21%), fetal growth restriction (19%), multiple pregnancies (12%), and preeclampsia (9%) in 57 neonates [16]. Several studies are in contrast with our data, reporting a higher incidence of early neonatal hypocalcemia in small-for-gestational-age neonates [25,26]. In a recent review article, Cheng et al. interpreted the pathophysiology of hypocalcemia in neonates with fetal growth restriction by placental pathology, particularly when associated with ischemia. Chronic fetal hypoxia and prolonged suboptimal nutritional supply lead to ineffective metabolic adaptations that impair appropriate intestinal absorption and bone resorption, leading to hypocalcemia [10,27,28].

Our study, to our knowledge, is the first to report that fetal growth restriction decreases the probability of early neonatal hypocalcemia. This study’s data expresses doubt about fetal growth restriction being a risk factor for early neonatal hypocalcemia in moderate and late neonates. The primary pathophysiologic mechanisms underlying fetal growth restriction are different; they often have the same final common pathway: suboptimal uterine–placental perfusion and fetal nutrition [29]. The etiology of many adverse consequences of fetal growth restriction arises in utero from fetal hypoxia and nutrient deprivation secondary to placental dysfunction [30]. Calcium homeostasis plays an important role during organogenesis under hypoxic conditions [31]. Calcium transport is important for placental functioning and fetal growth, and increased expression of placental calcium transporters was seen in pregnant rats exposed to 10–12% hypoxia [31,32,33]. Active placental calcium transport from mother to fetus in normal pregnancy maintains a higher calcium concentration in fetal compared to maternal plasma [34,35]. Fetal growth restriction is associated with complex, coordinated, and highly regulated changes in placental signaling and nutrient transport. Calcium ATPase actively pumps calcium into the fetal circulation [36,37]. It is important to highlight that basal membrane calcium ATPase activity is increased in fetal growth restriction [38]. The basal membrane calcium ATPase is up-regulated in fetal growth restriction, possibly due to elevated fetal concentrations of PTHrp 38–94, a key regulator of the placental calcium pump. That may represent a compensatory mechanism in response to an imbalance between calcium requirement and calcium supply [38,39,40]. These findings are not entirely consistent with the traditional view that the placenta is dysfunctional in fetal growth restriction but rather suggest that the placenta adapts to reduce fetal growth in response to an inability of the mother to allocate resources to the fetus [38]. With the loss of the placenta and the first breaths, serum calcium falls. The initial high level in fetal blood may reduce the likelihood that this obligatory fall in calcium causes symptomatic hypocalcemia [36]. It is possible that up-regulated calcium ATPase during fetal life could impact calcium levels during the first days of life in neonates with fetal growth restriction. Our data suggest a lower risk of early neonatal hypocalcemia in moderate and late term neonates with fetal growth restriction and speculate that fetal growth restriction could modulate the effect of perinatal hypoxia on neonatal calcium levels. Further studies are needed to confirm these results and elucidate the pathophysiology of early neonatal hypocalcemia in preterm neonates with fetal growth restriction.

To the authors’ knowledge, this is the first paper that includes cardiotocography—3-tiered fetal heart rate categorization—as a possible predictor of early neonatal hypocalcemia. A study revealed that 3-tiered fetal heart rate categorization, NICU admission acid base and blood gas analysis are not risk factors for the development of early neonatal hypocalcemia. The most likely explanation is that fetal heart rate patterns have poor positive predictive value for fetal compromise [41,42]. Vansprangles et al. showed no difference in umbilical cord calcium level in term neonates despite a statistically significant difference in acid base and blood gas [43]. On the contrary, Zanardo et al. reported a statistically significant positive correlation between calcium and lactate in arterial cord blood in healthy-term vaginally delivered neonates, pointing to a neonatal response to the stress of labor and delivery [44]. These data support complexity in calcium homeostasis in neonatal adaptation to extrauterine life.

In this study, we demonstrate that cesarean delivery is a risk factor for the development of early neonatal hypocalcemia. Bagnoli et al. measured statistically significantly lower serum calcium levels at birth and at 24 h of life in neonates born by cesarean delivery without labor compared to vaginal delivery, elective cesarean delivery with labor, and emergency cesarean delivery with fetal distress [45]. This finding is supported by Papandreou et al.’s study, where they demonstrated that total calcium levels in women and neonates undergoing spontaneous labor are significantly higher than in women undergoing scheduled cesarean section or women without clinical signs of initiation of labor [46]. In contrast with our findings, Jeong et al. reported that cesarean section was not a risk factor for early neonatal hypocalcemia [47]. We believe that cesarean delivery is an understudied risk factor for early neonatal hypocalcemia despite multiple indications for cesarean delivery in moderate and late neonates.

The overall rate of early neonatal hypocalcemia in our study was 36.5%. This aligns with data showing that approximately one-third of preterm infants have low serum calcium levels within the first 48 h of life [8,48]. Similar to our findings, Picone and Paolillo reported an incidence of hypocalcemia of 40.5% in a retrospective single-center study of 417 late preterm neonates, which predominantly occurred on the second and third day of life [49].

The overall rate of neonatal hypoglycemia in the first 24 h in our study was 31.3%, consistent with a recent cross-sectional study on 186 preterm neonates by Khan et al. [50]. Harris et al. described the incidence of hypoglycemia as 54% in late neonates, with 80% of hypoglycemia episodes occurring in the first 24 h [51]. In contrast to our results, Picone and Paolillo reported an incidence of hypoglycemia of 3% in late neonates, highlighting a difference in diagnostic criteria for neonatal hypoglycemia (<2.2 mmol/L) [49]).

In conclusion, we believe that our findings could contribute to the clarification of the risk factors for early neonatal hypocalcemia in the prevention of symptomatic clinical presentation of hypocalcemia.

### Strengths and Limitations of the Study

This was a retrospective case-control study, so it has limitations in establishing cause–effect relationships. The small number of neonates with fetal growth restriction, with only 24 neonates, is a major limitation of the study. We need to emphasize that the inclusion criteria for diagnosing early neonatal hypocalcemia cannot exclude the possibility of missed diagnoses of this condition. Phosphorus and magnesium levels have not been analyzed, and it is important to note that those electrolytes can impact calcium levels. Another possible limitation of the study is that we have not evaluated late-onset hypocalcemia. It is important to highlight the possible overlap of multiple causes of early neonatal hypocalcemia in our neonates due to the retrospective nature of the study. Larger prospective studies with long-term follow-up, particularly in preterm neonates with fetal growth restriction, are needed to establish changes in calcium levels after birth. We need to stress the possible difference in the timing of NICU admission among neonates, although most of the neonates were admitted to the NICU immediately after birth.

Our study is one of the few that recently analyzed fetal growth restriction as a risk factor for early neonatal hypocalcemia. Our findings speculate that cesarean delivery could be a risk factor for the development of early neonatal hypocalcemia. Despite limitations, to our knowledge, our study is the first to explore cardiotocography—3-tiered fetal heart rate categorization—as a possible risk factor for early neonatal hypocalcemia. The strength of the study is the definition of using base excess in extracellular fluid and HCO_3_ standard in blood gas determination. The major strength of our study is its population, which consists of moderate to late preterm neonates, a group that has been understudied until recently, as they are the most common type of preterm neonates.

## 5. Conclusions

The data in this study question whether fetal growth restriction is indeed a risk factor for early neonatal hypocalcemia in moderate and late preterm neonates. Cesarean delivery may be a risk factor for screening for early neonatal hypocalcemia in moderate and late neonates.

In conclusion, we believe that our findings contribute to the clarification of the risk factors for early neonatal hypocalcemia in the prevention of symptomatic clinical presentation of hypocalcemia.

## Figures and Tables

**Table 1:** Early neonatal hypocalcemia and hypoglycemia in FGR and control neonates.

	FGR (n = 24)	Control (n = 124)	*p* Value
Early neonatal hypocalcemia	1 (4.4%)	53 (42.8%)	<0.001 ^a^
Neonatal hypoglycemia	5 (20.8%)	35 (28.2%)	0.455 ^a^
Total serum Ca level (mmol/L)	2.178 (SD 0.180)	2.042 (SD 0.208)	0.004 ^b^
Serum glucose level (mmol/L)	3.350 (IQR 2.600–3.775)	3.000 (IQR 2.400–3.900)	0.334 ^c^

^a^ Chi-square test, ^b^ Student *t*-test, ^c^ Mann–Whitney U test.

**Table 2:** Admission acid–base and blood gas in FGR and control neonates.

	FGR (n = 24)	Control (n = 124)	*p* Value ^a^
pH	7.215 (7.173–7.258)	7.232 (7.190–7.280)	0.228
pCO_2_ (kPa)	7.086 (6.105–7.785)	6.720 (6.030–7.595)	0.660
HCO_3_ (mmol/L)	18.950 (17.125–20.600)	18.800 (17.400–20.375)	0.954
BE (mmol/L)	−7.050 (−8.950–(−4.200))	−5.950 (−8.100–3.900)	0.441
sO_2_ (%)	86.500 (80.600–91.550)	82.650 (76.875–89.850)	0.152

Venous blood sample. Results are expressed as median and interquartile range; ^a^ Mann–Whitney U test.

**Table 3:** Logistic regression of early neonatal hypocalcemia and maternal and neonatal factors.

		Parameter	t	*p*	95% CI
Model	(Constant)	0.497	8.159	0.002	
	Fetal growth restriction	0.037	9.679	0.001	1.29–5.65
	Mode of delivery	2.702	6.963	0.004	0.00–2.30
	Gestational age	0.920	0.210	0.323	0.64–1.31
	Birth weight	1.000	0.021	0.442	1.00–1.00
	Sex	0.563	2.254	0.067	0.27–1.19
	3-tiered fetal heart rate categorization	0.864	0.281	0.298	0.50–1.49
	pH	0.214	0.037	0.424	0–15.21
	pCO_2_	0.914	0.378	0.269	0.69–1.22
	HCO_3_	0.934	0.816	0.183	0.81–1.08
	BE(ef)	1.032	0.373	0.271	0.93–1.14
	Hypertensive disorders in pregnancy	2.977	1.435	0.116	0.50–17.73
	Diabetes mellitus in pregnancy	0.734	0.134	0.358	0.14–3.85
	PPROM * > 16 h	0.433	2.423	0.217	0.15–1.24

Cox and Snell R^2^ 0.149, Nagelkerke R^2^ 0.203, corrected classification 68.2%, * preterm premature rupture of membranes.

## Data Availability

The data presented in this study are available on request from the corresponding author due to privacy.

## Abbreviations

The following abbreviations are used in this manuscript:

FGR	Fetal growth restriction
FHR	Fetal heart rate
NICU	Neonatal intensive care unit
BE	Base excess
PTH	Parathyroid hormone
ATPase	Adenosine triphosphatase
